# *Bifidobacterium adolescentis* is intrinsically resistant to antitubercular drugs

**DOI:** 10.1038/s41598-018-30429-2

**Published:** 2018-08-09

**Authors:** Dhanashree Lokesh, Raman Parkesh, Rajagopal kammara

**Affiliations:** 1 Senior Research Fellow, Department of Protein Chemistry and Technology, CSIR-CFTRI, Mysore, 20 India; 2 Principal Scientist, Protein Science Center, Institute of Microbial Technology, Sector-39A, Chandigarh, India

## Abstract

Multiple mutations in the β subunit of the RNA polymerase (rpoβ) of *Mycobacterium tuberculosis (Mtb)* are the primary cause of resistance to rifamycin (RIF). In the present study, bifidobacterial rpoβ sequences were analyzed to characterize the mutations that contribute to the development of intrinsic resistance to RIF, isoniazid, streptomycin and pyrazinamide. Sequence variations, which mapped to cassettes 1 and 2 of the rpoβ pocket, are also found in multidrug-resistant *Mtb* (MDR *Mtb*). Growth curves in the presence of osmolytes and different concentrations of RIF showed that the bacteria adapted rapidly by shortening the growth curve lag time. Insight into the adapted rpoβ DNA sequences revealed that *B. adolescentis* harbored mutations both in the RIF pocket and in regions outside the pocket. The minimum inhibitory concentrations (MICs) and mutant prevention concentrations (MPCs) indicated that *B. longum, B. adolescentis* and *B. animalis* are resistant to antitubercular drugs. 3D-homology modeling and binding interaction studies using computational docking suggested that mutants had reduced binding affinity towards RIF. RIF-exposed/resistant bacteria exhibited variant protein profiles along with morphological differences, such as elongated and branched cells, surface conversion from rough to smooth, and formation of a concentrating ring.

## Introduction

The human gut microbiome consists of a vast number of microorganisms and is considered to be a bacterial ecosystem. The terms ‘gut microflora’ and ‘gut microbiota’ are used interchangeably, whereas ‘gut microbiome’ refers to the aggregate of all genomes within gut microbiota. The colon region of the human gut contains mainly anaerobic microbes, such as *Bifidobacterium* spp. The proportion of bifidobacteria is known to play a significant role in the maintenance of good health, but this role diminishes as the individual reaches adulthood^1^.

Bifidobacteria are thus considered to be one of the major health-promoting probiotic bacteria. The wisdom behind using antibiotic-resistant probiotic microbes is, as yet, unclear. One of the few commercial probiotics with significant antibiotic resistance, *Bacillus clausii*, has been extensively used/prescribed in Italy since the 1960s to treat viral diarrhea and antibiotic diarrhea (a side effect of antibiotic use) in children^2^. The use of antibiotic-resistant probiotics may help to preserve a population of useful bacteria in the bowel during antibiotic therapy. Otherwise, after antibiotic therapy, the gut microfilm may need to be reinstated. The great trouble with the use of antibiotic-resistant probiotics is the serious chance that horizontal gene transfer among gut microbes could create harmful antibiotic-resistant bacteria. It may therefore be safer to use only antibiotic-sensitive microbes as probiotics. The European Food Safety Authority (EFSA) report on Qualified Presumption of Safety (QPS) directs that the antibiotic status (resistance/susceptible) of microorganisms should be determined before approval^3^ (Opinion of the Scientific Committee on a request from EFSA on the introduction of a QPS approach for assessment of selected microorganisms referred to EFSA. The EFSA Journal, 587, 1–16. Available online: www.efsa.europa.eu/efsajournal). This means that antibiotic resistance *per se* is not considered to be a safety issue, but may be a concern if it leads to the transfer of resistance.

Tuberculosis (TB) continues to be a major global public health concern, with close to 9 million new cases and 1.5−2 million deaths each year^4^. The number of TB infections increases each year in developing and third world nations, primarily because of discontinuation of drug intake/inadequate treatment. Poor compliance also contributes to the emergence of drug-resistant *Mycobacterium tuberculosis* (*Mtb*). A report by Yumo *et al*. (2011) suggested that the number of new TB cases and deaths could be reduced by specific diagnosis and treatment^5^. *Mtb* strains that are resistant to isoniazid and rifamycin (RIF) are known as multidrug-resistant strains (MDR *Mtb*).

RIF and its second generation analogues (rifabutin, rifamixin and rifapentine) are the most potent and broad spectrum antibiotics available for treatment against MDR *Mtb*. These drugs are regarded to be equally important components of the most effective multidrug therapies for the treatment of virulent diseases, such as tuberculosis and brucellosis^6–8^. Previous reports suggested that the addition of RIF to culture media tends to modify cell morphology, leading to a ‘rough’ appearance^9^. Rough-surfaced *Brucella* are RIF-resistant and less virulent than RIF-sensitive strains^9,10^. These observations have led to the development of stable, rough, attenuated strains for use in vaccines against brucellosis. The rough surface of *Brucella* is used as a biomarker for suitability for vaccine production. The genetic basis of RIF resistance has also been thoroughly investigated in almost all prokaryotes/pathogenic microbes.

In the present study, we report, for the first time the morphology and mechanism of resistance of RIF-resistant Bifidobacterium spp. Several bifidobacterial strains were obtained from the German Collection of Microorganisms and Cell Cultures (DSM), and their resistance to different antitubercular drugs (RIF, isoniazid, streptomycin and pyrazinamide) was determined. We describe specific causes of RIF resistance in bifidobacteria and compare these microorganisms with *Escherichia coli* and MDR *Mtb*. The mechanistic aspects of Bifidobacterial resistance to RIF have not reported, and, this study is intended to be the foremost of its sort.

In prokaryotes, RIF targets the β subunit of the DNA-dependent RNA polymerase (RNAP), which is encoded by the rpoβ gene^11–15^. RIF shows very high binding affinity for the β subunit of RNAP and is thus a potent inhibitor of bacterial RNAP. Previous reports concluded that RIF binds to a pocket that contains cassettes I and II, termed the RIF pocket, in the β subunit of RNAP. Subsequently, it penetrates deep within the DNA-RNA channel of RNAP, blocking the path of the elongating RNA transcript at the 5′ end. Mutations in the RIF-binding pocket disrupt the confirmation of the binding pocket, creating distortions in the structure of rpoβ. This disrupts the binding of RIF, leading to the creation of a RIF-resistant microorganism^16,17^. The specific amino acid residues in cassettes I and II of the RIF pocket that are natural targets for mutagenesis have been identified. Recently, several missense mutations within the subunit have been characterized in RIF-resistant pathogenic bacteria, such as *E. coli*, *Brucella* and *Mtb*^18–24^.

Screening bifidobacterial strains obtained from the German Collection of Microorganisms and Cell Cultures (DSM) revealed resistance to different antitubercular drugs (RIF, isoniazid, streptomycin and pyrazinamide). RIF-resistance occurs due to two major mutated proteins, the β subunit of DNA-dependent RNA polymerase (rpoβ clusters 1,2 and 3) and ADP-ribosyltransferase^25^. The Bioinformatics information that in Bifidobacteria rpoβ and not ADP-ribosyltransferase is the significant constituent of the housekeeping gene^26^. So also *Bifidobacterium adolescentis* lacked the ortholog of the ADP-ribosyltransferase (Based on Biocyc database, https://biocyc.org/) revealed a role for rpoβ in Bifidobacteria RIF-resistance resulting in different cell morphology. This paper describes mechanistic aspects of Bifidobacterial resistance to RIF.

The study involved the isolation and identification of different bifidobacteria with mutations in rpoβ associated with the RIF-resistant phenotype. The resistant bacteria were further characterized by determining their susceptibility to isoniazid, RIF, streptomycin and pyrazinamide. The particular rpoβ region that encodes cassette I and II (rpoβ pocket and also the adjacent region) were amplified, sequenced and analyzed. Finally, the results were compared with those of *E. coli* and MDR *Mtb* rpoβ cassettes I and II, and the adjacent regions. To understand the affinity among rpoβ, mutants (mutations at cassettes 1, and 2, even in the adjacent regions; known as mutant 1 and mutant 2) and RIF, structural biology studies (such as modeling and docking) were also conducted.

## Results

**Figure 1:**
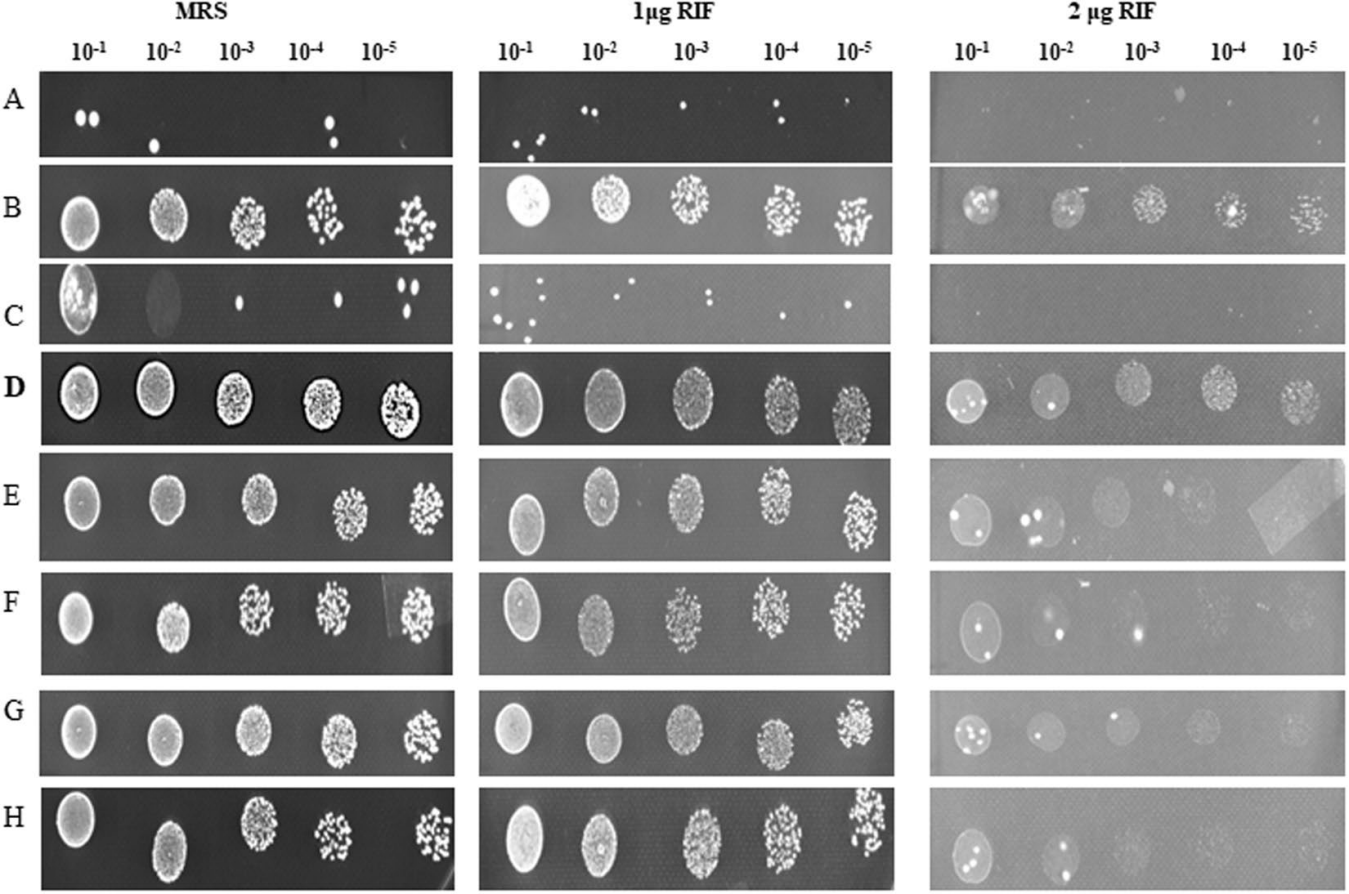
RIF resistance of different Bifidobacteria (Bold represents *B. adolescentis*). (**A**) 20219: *B. longum subspp longum* (**B**) 15837: *B. thermoacidophilum subspp thermoacidophilum* (**C**) 20089 (**D**) **20083:** ***B. adolescentis*** (**E**) 20088: *B. longum subspp infantis* (**F**) 20213: *B. breve* (**G**) 10140: *B. animalis subspp. lactis* (**H**) 20105*: B. animalis*.

**Figure 2:**
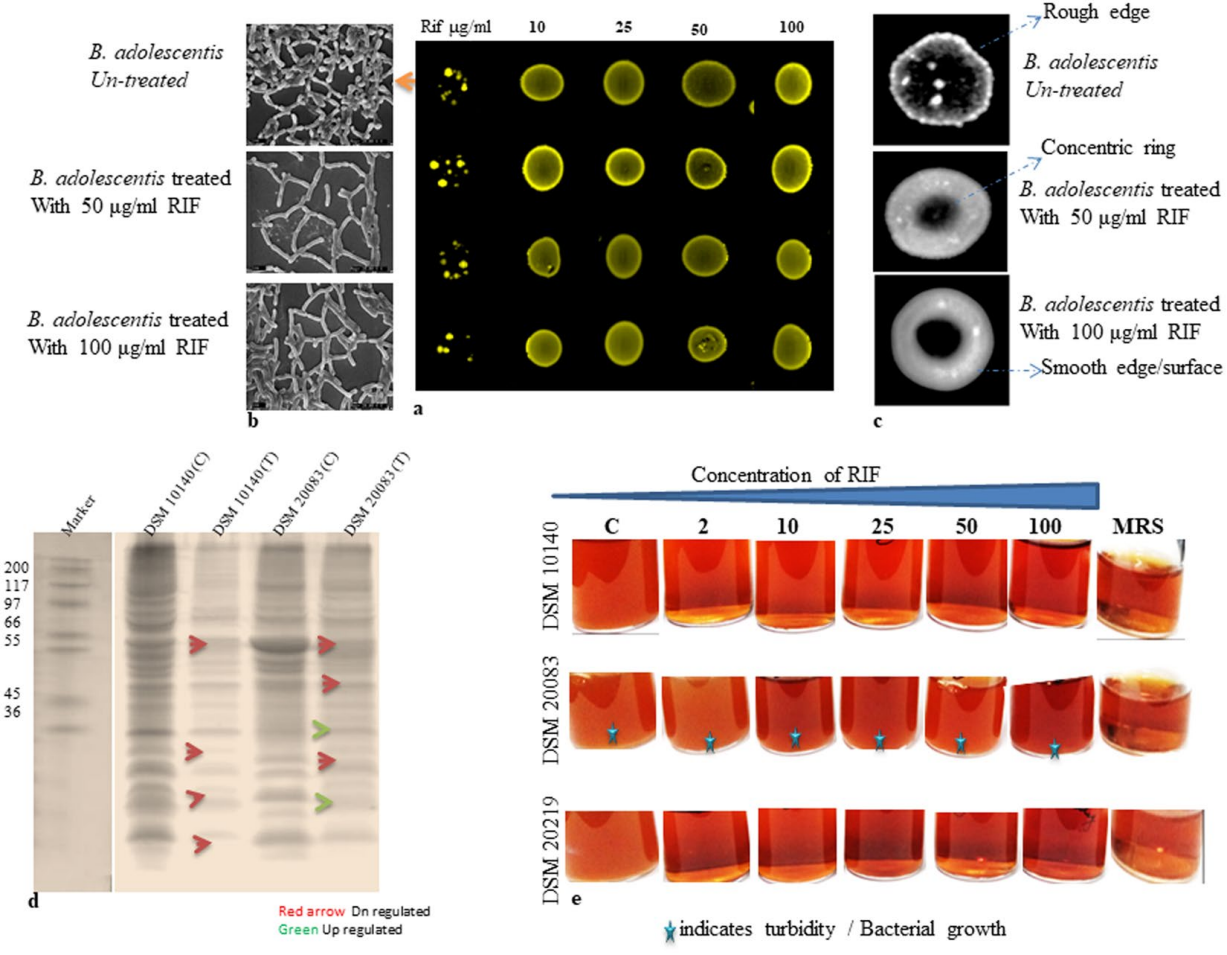
(**a**) Spot assay to understand RIF resistance in *B. adolescentis* (**b**). SEM studies (**c**). Compound microscope of *B. adolescentis* (**d**). Protein Profile (**e**). Resistance in terms of viability.

To investigate antitubercular drug resistance and alterations to the rpoβ sequence, *B. animalis, B. longum and B. adolescentis* were cultured in a 96-well plate. All strains of bifidobacteria were subjected to a MIC assay, spot assay, and cell viability assay (Table 1, Figs 1 and 2a,b), and the development of resistance was observed (Table 1). There was a difference in the resistance observed among these organisms (Fig. 1), demonstrating that bifidobacteria were more immune to drugs, such as RIF, pyrazinamide, isoniazid, and streptomycin, used in multidrug combinations (Table 1). All of the bifidobacteria used in the study showed very high levels of resistance to pyrazinamide, isoniazid, and streptomycin (>200 μg/ml). *B. adolescentis* showed very high resistance to RIF (>1.56 μg/mL, Fig. 2a,b and Tables 1 and 2). *B. animalis* grew very slowly, and antibiotic resistance was considered to be much lower than in the other bifidobacteria strains. Therefore, *B. animalis* was not included in further studies. *B. longum* grew at a rate similar to that of *B. adolescentis*, but the resistance was not profound. We next conducted growth curve studies of all selected bifidobacteria strains in medium containing RIF (2 μg/mL) together with osmolytes. The RIF resistance of *B. adolescentis* was further confirmed using the spot assay (Fig. 2a), in which *B. adolescentis* showed resistance at different dilutions that were spotted onto RIF-containing agar plates. All of the bifidobacteria strains were subjected to a spot assay (Fig. 1), but only *B. adolescentis* was considered to show considerable resistance to RIF (Figs 1 and 2a,b). The remaining strains either did not grow or had imperceptible growth, indicating their susceptibility to RIF. Viability studies were conducted to investigate the existence of resistant colonies (Fig. 2b). *B. animalis* and *B. longum*, but not *B. adolescentis*, were susceptible to RIF (Table 2 and Fig. 2). These studies further confirmed the intrinsic resistance of *B. adolescentis* to RIF. Having demonstrated the resistance of *B. adolescentis* to RIF, we next conducted MTT assays with different concentrations of RIF to investigate cell viability.

**Table 1:** MIC of various bifidobacteria with antitubercular drugs.

Bacterial strain	Strain no	MIC (ug/ml)^a^ of the following antibiotic^b^ for the indicated organism			
		RIF	PYR	INH	SM
*B. animalis subspp. lactis*	DSM 10140	0.78	>200	200	50
*B. adolescentis*	DSM 20083	1.56	>200	>200	100
*B. longum subspp. longum*	DSM 20219	0.78	>200	>200	100
*Mycobacterium tuberculosis* H37Rv^c^	ATCC27294	0.8^c^	NA	0.2^c^	5^c^

^a^Median of the 2 repetitions.

^b^RIF,Rifampicin; PYR, Pyrazinamide; INH, Isoniazid; SM,Streptomycin sulphate.

^c^Reference -Jhamb, Sarbjit Singh, Amit Goyal, and Prati Pal Singh. “Determination of the activity of standard anti-tuberculosis drugs against intramacrophage Mycobacterium tuberculosis, *in vitro*: MGIT 960 as a viable alternative for BACTEC 460.” *Brazilian Journal of Infectious Diseases* 18, no. 3 (2014): 336–340.

NA: Not available.

Bifidobacteria were grown in the presence of RIF, pyrazinamide, streptomycin and isoniazid. Serial dilutions of the drugs were made directly in a sterile 96-well flat bottom microtitre plate containing MRS medium (100 μL). The RIF concentration range was 0.02–200 μg/mL. ^a ^Median of the two repetitions.

**Table 2:** Resistance in terms of viability.

Conc (µg/ml)	DSM 10140	DSM 20083	DSM 20219
2	−0.49	0.815 ± 0.162	−0.51
10	−0.65	0.76 ± 0.028	−0.461
15	−0.428	1.06 ± 0.084	−0.193
50	−0.98	1.243 ± 0.046	−0.193
100	−0.12	0.705 ± 0.106	−0.461

*B. adolescentis, B. animalis and B. longum* were grown in the presence of different concentrations of RIF (2–100 µg/mL). The cells were withdrawn, spread on a plate and counted.

SEM images of *B. adolescentis* grown in the presence of RIF (50 and 100 μg/mL) showed elongated and heavily branched bacteria with fewer small hairy structures and terminal buttons/hyphae compared with the control. When the same cells were exposed to RIF (100 μg/mL), the only changes were reduced hairy structures (Fig. 2b). Surface analysis of these cells showed that ‘concentric’ circles began to form when the concentration of RIF was 50 μg/mL (Fig. 2c). The colony was milky white, and a rough surface edge was noted. Upon exposure to a higher concentration of RIF (100 μg/mL), the ‘concentric’ circles were more visible and enlarged, and the bacterial edges were very smooth and milky white. These concentric circles were absent in the parent colonies. The protein profile of the cells exposed to RIF was determined by isolating the membrane proteins and separating them using SDS-PAGE (Fig. 2d). A large number of proteins were either downregulated (66, 55 and 25 kDa) or upregulated (~25–30 kDa) in *B. adolescentis*. Surprisingly, there was no protein upregulation in the case of *B. animalis* and only gene downregulation occurred (Fig. 2d). It was further noted that only low molecular weight proteins were upregulated, whereas the downregulated proteins had higher and also lower molecular weights.

After exposure of the microbes to RIF, the roughness or smoothness of the cell wall structures was analyzed by binocular observations. Cells grown in the presence of different concentrations of RIF were isolated and analyzed by SEM, compound microscopy (Fig. 2b,c) and protein profiling (Fig. 2b–d). Viability studies shown in Fig. 2e clearly demonstrate that only *B. adolescentis* shows considerable resistance to RIF. The growth of three different bifidobacterial strains in the presence of different concentrations of RIF, together with the results of the spot and viability assays, is shown in Fig. 2a,e. Viability was further confirmed by the MTT assay, in which only DSM 20083 showed resistance to all concentrations tested (Table 2, Fig. 2e). The considerable amounts of turbidity in DSM 20083 show a difference in growth between the control bacteria and those exposed to different concentrations of RIF (*B. adolescentis*, Fig. 2e middle row). Substantially less growth or negligible growth were observed for the remaining strains as the RIF concentration increased from 2–100 µg (Fig. 2e DSM 10140 and DSM 20219, Fig. 2e 1^st^ and 3^rd^ rows).

The resistance of *Bifidobacterium* spp. strains to RIF was investigated in detail by performing growth curve studies in the presence or absence of various concentrations of the drugs/osmolytes. Growth curve studies of *B. animalis* (DSM 10140), *B. adolescentis* (DSM 20083) and *B. longum* (DSM 20219), conducted at an OD_600_ of 0.01 in the presence or absence of osmolytes, showed that the lag time was markedly reduced by exposure to RIF. Of the three *Bifidobacterium* spp. strains chosen for growth curve studies in the presence of RIF (2 µg/mL), only *B. adolescentis* was considered suitable for further antibiotic resistance studies, although all of the strains showed resistance to all of the antitubercular drugs over the concentration range 0.2–200 μg/mL. *B. animalis subspp. lactis* (DSM 10140) and *B. longum subspp. longum* (DSM 20219) showed minimal resistance to RIF (both 0.78 μg/mL) (Table 1), whereas *B. adolescentis* showed considerably high resistance to RIF (>1.56 μg/mL) and was, therefore, considered for further studies.

**Figure 3:**
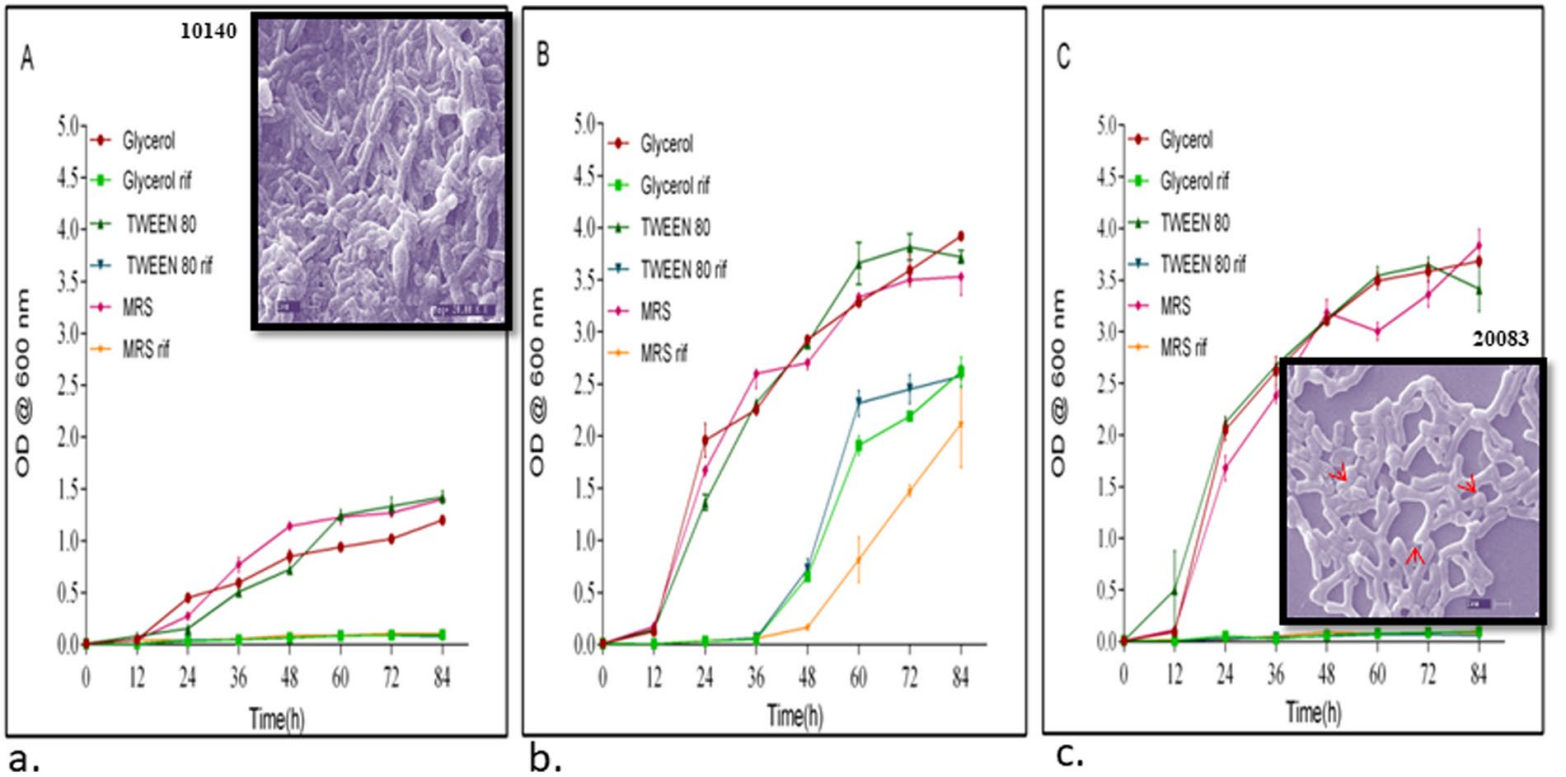
(**a**–**c**) inset SEM of RIF treated cells (Inset (**a**) 10140, (**c**) 20083) Arrows in (**c**) indicate terminal hairy structures.

The growth curves (Fig. 3a–c) showed that the addition of the osmolytes, glycerol, and Tween 80 did not hamper bacterial growth. The presence of RIF had a profound effect on growth. *B. adolescentis* showed a lag time of 36–48 h, but later growth returned to a normal rate. In the presence of MRS + RIF, the lag time was much greater in the case of *B. adolescentis*, but the delay time was reduced after the introduction of Tween 80 or glycerol. The principal function of osmolytes is to permeabilize the cell wall, leading to active uptake of the drug. In the present study, the lag time in the growth curve studies with RIF was reduced by the introduction of the osmolytes, confirming the ability of bifidobacteria to adapt to RIF. The lag time was very high (48 h) in the case of *B. adolescentis* exposed to MRS + RIF in the absence of osmolytes. The remaining bifidobacteria strains did not grow well in MRS + RIF but were viable. Further studies confirmed that RIF inhibited the growth of both DSM 20219 and DSM 10140. During the growth curve analysis, small amounts of DSM 10140 and 20083 were subjected to SEM analysis. It was found that the cells were elongated, hairy and rough surfaced (Fig. 3a inset). In the case of *B. adolescentis*, a smooth surface with terminal hairs (Fig. 3c inset, red arrows indicates hairy structures) was observed. No considerable changes were observed among the other bifidobacteria strains.

**Figure 4:**
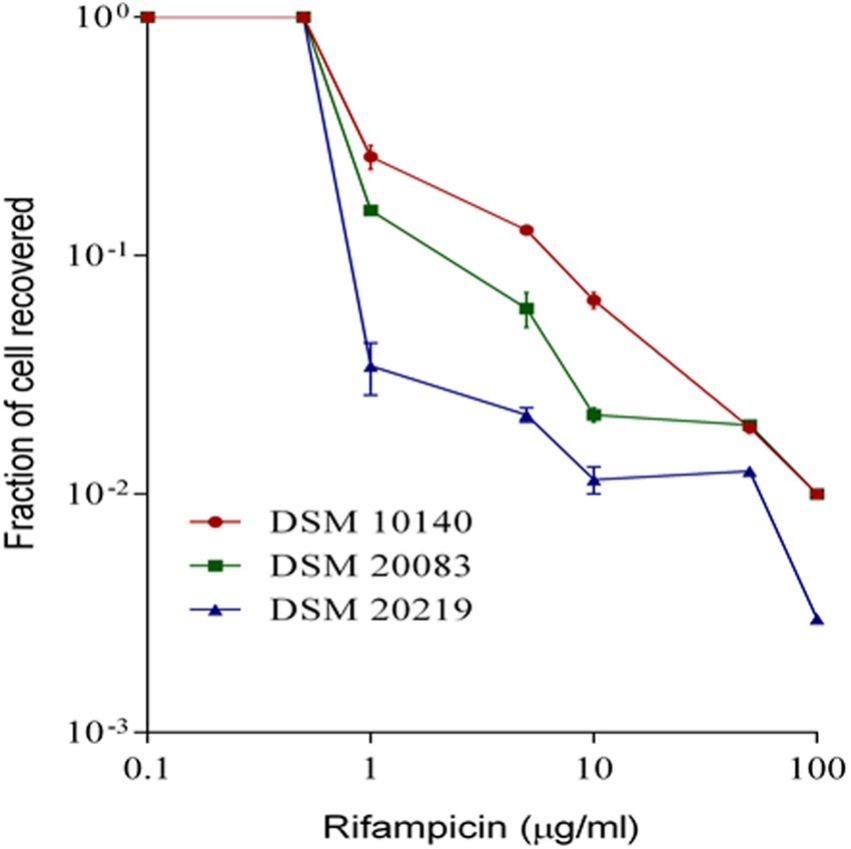
Mutant Prevention Concentration of various Bifidobacteria.

It is possible that the resistance of DSM 20219 and DSM 10140 is a transient or pseudo resistance. To investigate this possibility, we conducted viability/survival studies on the same RIF-treated cells. It is acknowledged that continuous exposure of bacteria to an antibiotic creates resistance. To investigate the hypothesis, we carried out an MPC (mutant prevention concentration) assay (Fig. 4) to overcome the problem of variability among the MIC values. In the present study, all three bifidobacteria (10^10^ CFU/ml stationary phase cells) were incubated with different concentrations of RIF, ranging from 0–100 µg/mL. Resistance was observed among all bifidobacteria (Fig. 2a), but the number of strains showing RIF resistance decreased as the RIF concentration reached 100 μg/mL (Fig. 2e). The spot assay with *B. adolescentis* further demonstrated RIF resistance at concentrations over the range 10–100 μg/mL (Fig. 2a). The spot assay showed that bifidobacteria incubated with 10 µg/mL RIF grew very well, even on the 100 µg RIF plate (Fig. 2a). The growth of cells incubated with other concentrations of RIF, and their subsequent growth at higher concentrations of RIF, are shown in the Fig. 2a.

### RIF uptake and DNA sequencing

**Figure 5:**
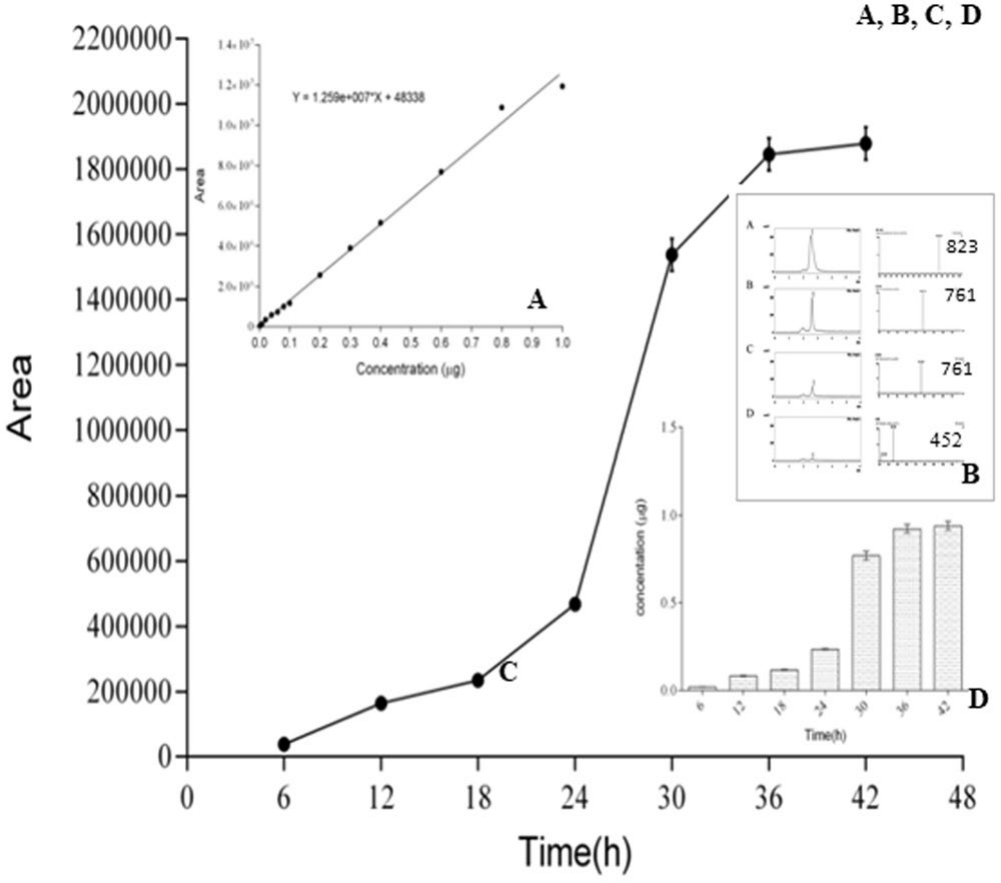
RIF uptake.

Uptake studies using HPLC and LC-MS were also conducted to quantify the amount of RIF consumed by the bifidobacteria over time. Before investigating RIF uptake, we prepared a standard curve for RIF (Fig. 5 inset A). Cell lysates prepared from bifidobacteria exposed to RIF for different lengths of time were extracted and injected into an HPLC apparatus. The eluted peaks were subjected to LC-MS. The HPLC and LC-MS peaks (*m/z* 761 and 452) are shown in Fig. 5 inset B. Most peaks had lower molecular weights and did not correspond to RIF (Fig. 5B inset A, B, C, D) likely because the bifidobacterial system can metabolize any RIF uptake (Fig. 5 inset B). Further, Fig. 5B shows that the peaks were very different, especially in Fig. 5B inset D, and the molecular weight of the compound was much lower than that of RIF. There is also a possibility that the low molecular weight products may be degradation products of RIF. Przybylski *et al*.^27^ reported that fragmentation of RIF leads to the formation of many aza-analogues, which may explain the products observed in our study. Surprisingly, the RIF fragments at *m/z* 761 and 452 were not observed in the mass spectrum. Therefore, the metabolites may be highly unstable and having short half-lives, might have been degraded. There is a possibility that bifidobacteria may have a mechanism for metabolizing RIF, which needs to be investigated further.

To quantify RIF uptake by bifidobacteria, the peak area was plotted against time (Fig. 5C). The graph shows the absorption of RIF, and the inset figure illustrates the increase in uptake as the incubation time is increased (Fig. 5D). Maximum uptake was observed after incubation of the cells with RIF for 36–42 h. The histogram (Fig. 5D inset) provides clear cut information. The control standard curves for different concentrations of RIF are shown in Fig. 5A. To verify and further illustrate bacterial resistance to RIF, the cells were grown in the presence of different concentrations of RIF (10–100 µg/mL). The control was grown in MRS medium in the absence of RIF. All the cells were subsequently spotted onto the RIF plate as described previously.

**Figure 6:**
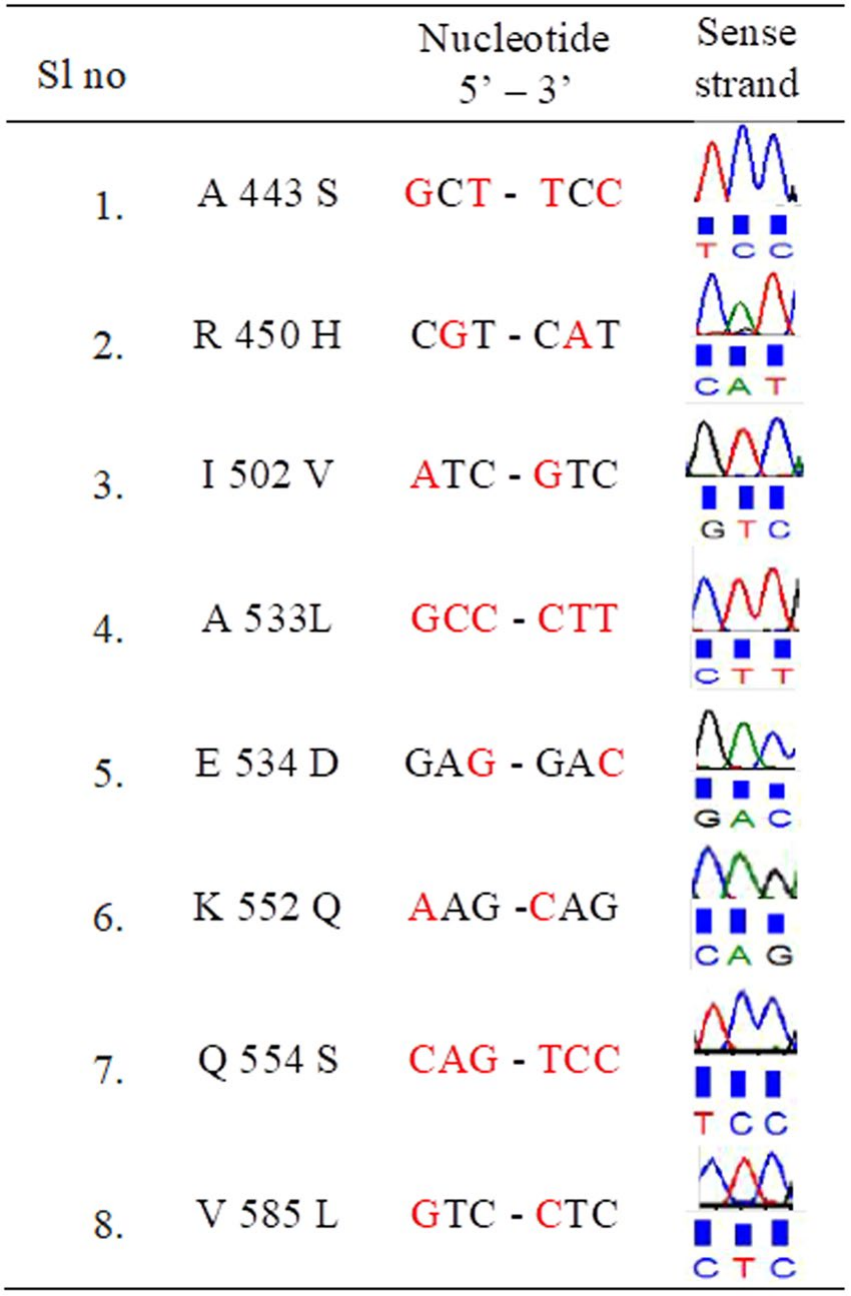
RIF pocket of the Bacteria which were treated with various concentrations (2–100 µg) of RIF was sequenced. Mutations were determined by comparing with the sequence of the untreated sample. Amino acid and its number, nucleotide change and corresponding chromatogram in sense direction are tabulated.

**Figure 7:**
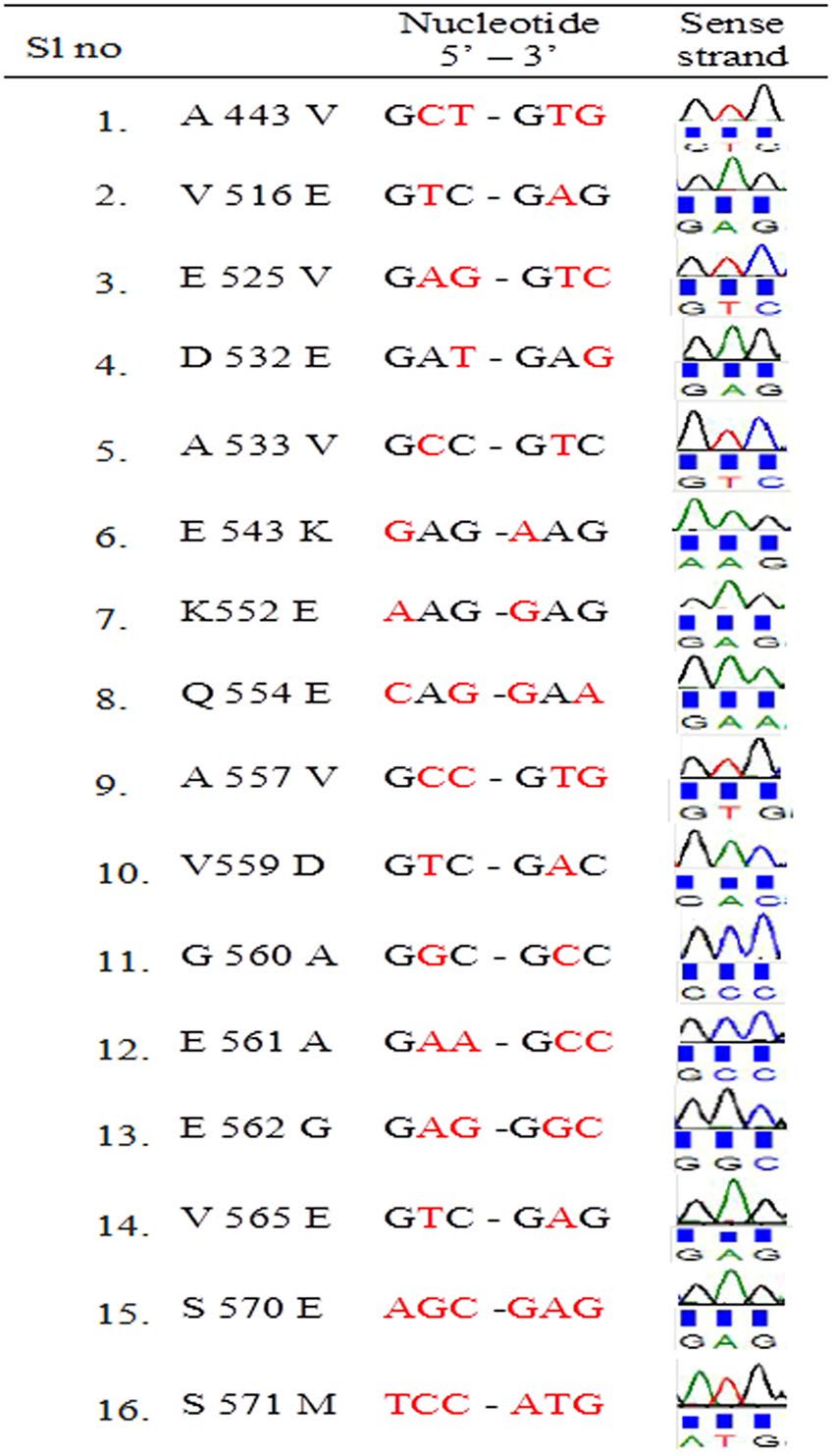
RIF pocket of the Bacteria which were treated with various concentrations (2–100 µg) of RIF was sequenced. Mutations were determined by comparing with the sequence of the untreated sample. Amino acid and its number, nucleotide change and corresponding chromatogram in sense direction are tabulated.

Cells that showed adaptability were subjected to PCR amplification of the rpoβ gene. All amplified products were of similar molecular weights (~750 bp, data not shown). Bifidobacteria incubated with different concentrations of RIF were also subjected to DNA sequencing to investigate mutations in the rpoβ gene. The DNA sequencing results demonstrated mutations, even in wild type bifidobacterial rpoβ, that are precisely the same as those observed in MDR *Mtb*. Many other variations were found, both in the rpoβ cassettes and in other regions. These observations demonstrate that intrinsic rpoβ mutations cause antitubercular drug resistance in bifidobacteria (Figs 6 and 7).

Wild type *B. adolescentis* (DSM 20083) did not show any mutations in the clusters/hot spot regions, and the sequence was exactly the same as that of H37RV. Mutations (valine to isoleucine, valine to glutamic acid, aspartic acid to glutamic acid and leucine to valine) were, however, observed in other regions (Figs 6 and 7). Several more mutations were observed in and near the cluster 3, but not in the clusters.

### Adaptation experiments

*B. adolescentis* was incubated with different concentrations of RIF (2, 15, 25 and 100 μg/mL), and the rpoβ cluster regions were subjected to DNA sequencing. Incubation with 2, 15 and 25 μg/mL RIF resulted in mutations in cluster 1 (alanine to serine or valine and arginine to histidine). When the cells were subjected to a higher concentration of RIF (100 μg/mL), however, serine (which was originally alanine in the wild type) was replaced by valine. No variations were discovered in clusters 2 and 3. A larger number of mutants were noted in the other regions between clusters 2 and 3. These mutations included valine to glutamic acid, glutamic acid to valine, aspartic acid to glutamic acid, leucine to valine and aspartic acid to glutamic acid. The conserved region, VGEE, observed between clusters 2 and 3, was unchanged upon exposure to lower concentrations of RIF (2, 15 and 25 μg/mL) but was mutated to DAAG upon treatment with a higher concentration of RIF (100 μg/mL). In the serine-rich region following the conserved region, serine residues were replaced by glutamic acid and methionine residues. The largest number of mutations was observed in the region between clusters 2 and 3, but not in any of the cluster areas (Figs 6 and 7).

### Bifidobacteria adaptation to a higher concentration of RIF

Wild type bifidobacteria that developed resistance upon exposure to 2 µg/mL RIF were subjected to higher concentrations of RIF to investigate the nature of the adaptation. Bifidobacteria adapted by rapidly changing their rpoβ sequences. The mutations observed in rpoβ clusters 1 and 2 are shown in Figs 6 and 7. These DNA sequence results were aligned with that of MDR *Mtb* (Table 3). The rapidly adaptable behavior of bifidobacteria towards RIF could also be seen in the reduced lag time in the growth curves (Fig. 3a–c). Astonishingly, significantly large numbers of different mutations were observed in regions other than the hot spots. These mutations have not been previously described, even in MDR *Mtb*. It is possible that these mutations are responsible for the adaptable nature of bifidobacteria. Upon exposure to stepwise increases in RIF concentration, the microbes slowly began adaptation. Further characterization of these mutations may reveal their importance in the adaptation to higher concentrations of RIF. The morphology (rough or smooth) of bifidobacteria that were subjected to different concentrations of RIF was subsequently investigated^28^. The surface of wild type bifidobacteria, which is known to take up crystal violet, is considered to be rough. The surface of RIF-treated bifidobacteria, on the hand, was smooth, indicating that it will not take up crystal violet.

**Table 3:** Multiple sequence alignment of MTB (H37RV, MDR, and XDR) with DSM 20083 (Control and 2 µg, 15 µg, 25 µg, and 100 µg RIF treated samples).

H37RV MT	INIRPVVAAI**KEFFGTSQLSQFM**D**QNNPLSGLTHKRRLSA**L**GPGGLS**RERAGLEVRDVHP	
MDR MTB	INIRPVVAAI**KEFFGTSQLSQFMYQNNPLSGLTHKRRLSALGPGGLS**RERAGLEVRDVHP	
XDR MTB	INIRPVVAAI**KEFFGTSQLSQFMGQNNPLSGLTHKRRLSAPGPGGLS**RERAGLEVRDVHP	
DSM 2008	INIRPVNATI**KEFFGTSQLSQFMDQNNPL**A**GVTNKR**R**LSALGPGGLS**RDRASMEVRDVHP	
2 µg DSM 20083	INIRPVNATI**KEFFGTSQLSQFMDQNNPLSGVTNKRHLSALGPGGLS**RDRASMEVRDVHP	
15 µg DSM 20083	INIRPVNATI**KEFFGTSQLSQFMDQNNPLSGVTNKRHLSALGPGGLS**RDRASMEVRDVHP	
25 µg DSM 20083	INIRPVNATI**KEFFGTSQLSQFMDQNNPLSGVTNKRHLSALGPGGLS**RDRASMEVRDVHP	
100 µg DSM 20083	INIRPVNATI**KEFFGTSQLSQFMDQNNPLVGVTNKRRLSALGPGGLS**RDRASMEVRDV**P**P	
H37RV MTB	SHYGRMCPI*ETPEGP*N*IGLIGSL*SVYARVNPFGFIETPYRKVVDGVVSDEIVYLTADEED	
MDR MTB	SHYGRMCPI*ETPEGP***S***IGLIGSL*SVYARVNPFGFIETPYRKVVDGVVSDEIVYLTADEED	
XDR MTB	SHYGRMCPI*ETPEGPNIGLIGSL*SVYARVNPFGFIETPYRKVVDGVVSDEIVYLTADEED	
DSM 20083	SHFGRMCPI*ESPEGPNIGLIGSL*ATFGRINPFGFIETPYRKVVNGHVTDEVEYMTADRDA	
2 µg DSM 20083	SHFGRMCPI*ESPEGPNIGLIGSL*ATFGR**V**NPFGFIETPYRKVVNGHVTDEVEYMTADRD**L**	
15 µg DSM 20083	SHFGRMCPI*ESPEGPNIGLIGSL*ATFGR**V**NPFGFIETPYRKVVNGHVTDEVEYMTADRD**L**	
25 µg DSM 20083	*SHFGRMCPI* *ESPEGPNIGLIGSL* *ATFGR* ***V*** *NPFGFIETPYRKVVNGHVTDEVEYMTADRD* ***L***	
100 µg DSM 20083	SHFGRMCPI*ESPEGPNIGLIGSL*ATFGRINPFGFIETPYRKV**E**NGHVTDEV**V**YMTADR**EV**	
H37RV MTB	RHVVAQANSPIDADGRFVEPRVLVRRKAGEVEYVPSSEVDYMDVSPRQMVSVATAMIPFL	
MDR MTB	RHVVAQANSPIDADGRFVEPRVLVRRKAGEVEYVPSSEVDYMDVSPRQMVSVATAMIPFL	
XDR MTB	RHVVAQANSPIDADGRFVEPRVLVRRKAGEVEYVPSSEVDYMDVSPRQMVSVATAMIPFL	
DSM 20083	EHVIAQANQELDENGNFVKKQALARVGEEEAVDVPVSSVDYMDVSPRQMVSVGASLIPFL	
2 µg DSM 20083	**D**HVIAQANQELDENGNFV**Q**K**S**ALARVGEEEAVDVPVSSVDYMDVSPRQMVS**L**GASLIPFL	
15 µg DSM 20083	**D**HVIAQANQELDENGNFV**Q**K**S**ALARVGEEEAVDVPVSSVDYMDVSPRQMVS**L**GASLIPFL	
25 µg DSM 20083	**D**HVIAQANQELDENGNFV**Q**K**S**ALARVGEEEAVDVPVSSVDYMDVSPRQMVS**L**GASLIPFL	
100 µg DSM 20083	EHVIAQANQ**K**LDENGNFV**E**K**E**AL**V**R**DAAG**EA**E**DVPV**EM**VDYMDVSPRQMVSVGASLIPFL	
H37RV MTB	EHDDANRALMGANMQRQAVPLV	• DSM 20083 and Mtb(H37RV, MDR, XDR) amino acid residues
MDR MTB	EHDDANRALMGANMQRQAVPLV	
XDR MTB	EHDDANRALMGANMQRQAVPLV	• **CLUSTER1(Bold underlined)**
DSM 20083	EHDEGHRALMGTNMQRQAVPLI	• *CLUSTER2 (Italic underlined)*
2 µg DSM 20083	EHDEGHRALMGTNMQRQAVPLI	• CLUSTER3 (underlined Black)
15 µg DSM 20083	EHDEGHRALMGTNMQRQAVPLI	• Control (aerial font)
25 µg DSM 20083	EHDEGHRALMGTNMQRQAVPLI	• **Mutated (Bold underlined)**
100 µg DSM 20083	EHDEGHRALMGTNMQRQAVPLI	• Other than a cluster

### Homology modeling and Docking studies

**Figure 8:**
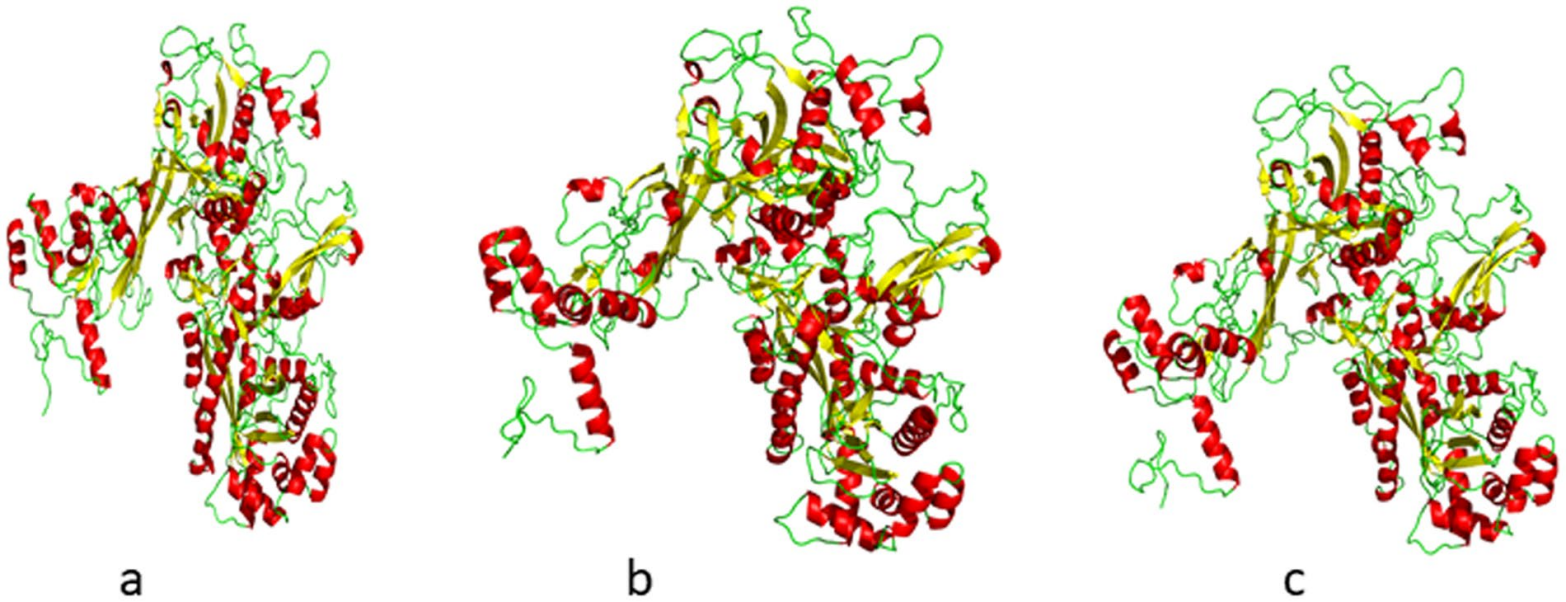
(**a**) 3D-homology model of rpo*β*. (**b**) 3D-homology model of mutant 1 rpo*β*. (**c**) 3D-homology model of mutant 2 rpo*β*.

**Figure 9:**
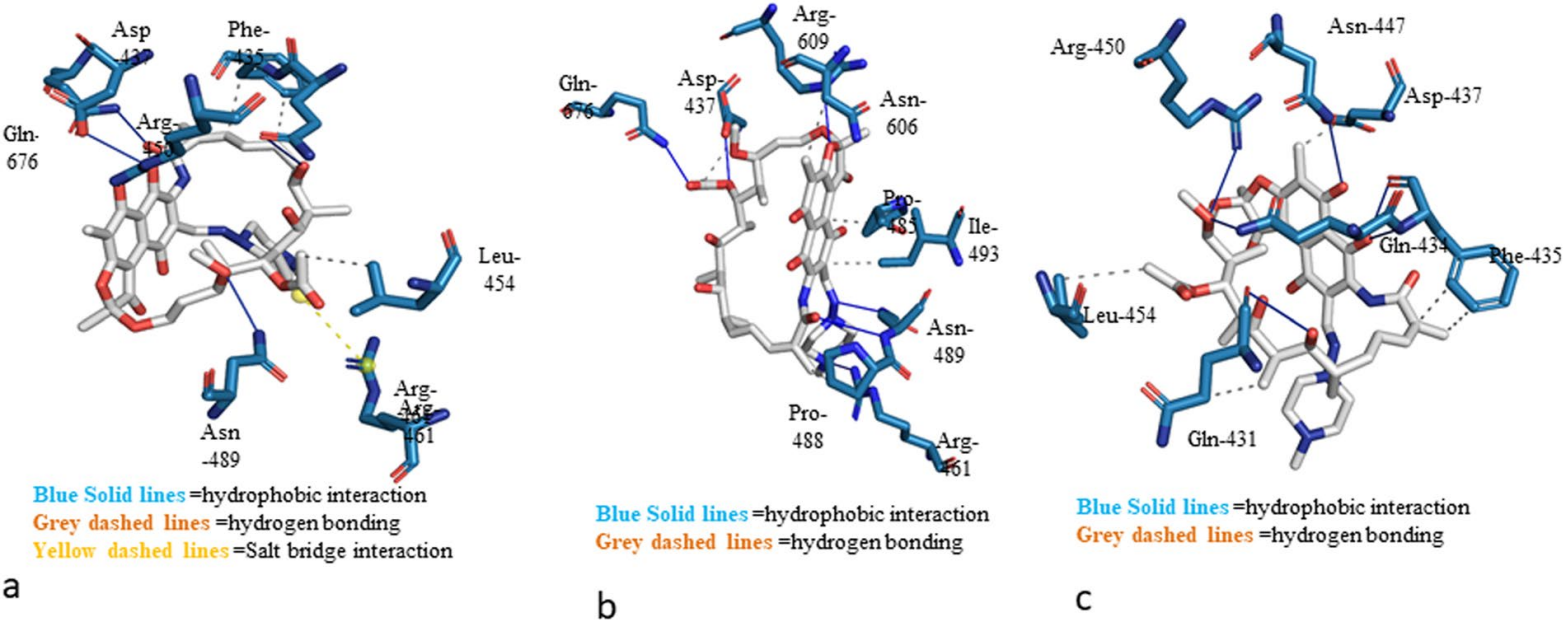
(**a**) Docked pose of RIF with wild type rpoβ BIFAA. (**b**) Docked pose of RIF with mutant 1 rpoβ BIFAA. (**c**) Docked pose of RIF with mutant 2 rpoβ BIFAA.

To explore the structural features of rpoβ BIFAA, a homology model was generated (Fig. 8a). This modeling involved the alignment of the target rpoβ BIFAA sequence with the known three-dimensional structural templates. Modeler 9.1 with template PDBs (PDB ID:5uh, 5vi8) was used to generate these homology models. We used residues from 25 to 1154 to generate the homology model of the wild-type and mutant proteins based on secondary structure analysis. The three-dimensional model of the target sequences containing all of the main and side chain non-hydrogen atoms was generated (Fig. 8a–c). For wild-type as well as mutant proteins, the three-dimensional models were generated, and the best model was selected based on the DOPE score for further analysis. The Ramachandran plot showed that the overall stereochemical quality of the generated models was good. The Ramachandran plot for wild and mutant model showed that almost all of the residues were in the most favored, additionally allowed, or generously allowed regions (data not shown). The docking studies are shown in Fig. 9a,b,c.

## Discussion

Sherpa *et al*.^29^ proposed that the formation of antibiotic-resistant microbes is not only a natural but also an evolutionary process. A few probiotic strains, including *Lactobacillus* and *Enterococcus* spp., have been reported to be antibiotic-resistant. In one way, acquisition of antibiotic resistance is thought to be disadvantageous since the resistance may spread through horizontal or vertical gene transfer in a natural and long-term process. This would argue that individual probiotic microbes should be tested for antibiotic-resistance markers before marketing, although, as of today, no agency has taken a lead in this. On the other hand, if concomitant antibiotic and probiotic therapy is needed, antibiotic-resistant probiotic bacteria may be seen as advantageous^30,31^. Neither Hammad and Shimamoto^30^ nor Varankovich *et al*.^31^ were, however, able to demonstrate the efficacy of combined probiotic-antibiotic therapy. The question of intrinsic and acquired resistance arises if the gene coding for resistance has been acquired and introduced into either the plasmid or the genome. Confusion arises if the mutations acquired in the long-term by probiotic microbes are in housekeeping genes, such as rpoβ. Such events have not been reported, but would be serious. Resistance to antitubercular drugs, as in the case of MDR *Mtb*, is considered to be the most dangerous situation. There are, however, no reports describing antitubercular drug-resistant probiotic microbes or the process by which they might acquire such resistance. Previous studies on antibiotic resistance in bifidobacteria by Wei *et al*., Gueimonde *et al*., and Varankovich *et al*. concluded that a few strains of bifidobacteria are intrinsically resistant to ciprofloxacin, nalidixic acid, mupirocin, streptomycin and aminoglycosides^32–34^. Few researchers subsequently identified antibiotic/aminoglycoside-resistant bifidobacteria^35–37^, but to date, RIF-resistant bifidobacteria have not been described. In the present report, for the first time, we have identified very highly RIF-resistant bifidobacteria.

Here, we have demonstrated that *B. adolescentis* shows resistance to RIF at very high concentrations and to an extent greater than the resistance for MDR *Mtb*. The resistance was shown to be caused by variations in rpoβ, which is an essential housekeeping gene that plays a significant role in protein synthesis. The present study also showed that bifidobacteria can quickly adapt to the antibiotic RIF. This phenomenon suggests that *B. adolescentis* may still be the best probiotic microbe, since it can quickly adapt to the antibiotic environment.

Our studies (MIC, MTT/viability assay, Spot assay, MPC and growth curve studies) also show that *B. adolescentis* has the ability to withstand, and grow in, very high concentrations of RIF. It was also observed that RIF uptake is possible since it was identified and measured in the bacterial lysate. Current antitubercular therapy consists of RIF, pyrazinamide, streptomycin and isoniazid. It may also be envisaged that *B. adolescentis* could be used safely as a probiotic since the rpoβ gene is a housekeeping gene present in almost all prokaryotes and therefore, may not be subject to horizontal gene transfer. The use of probiotics to address the problem of emerging antibiotic resistance may not be, as suggested by Imperial and Urbana, a double edged sword^38^, and there could certainly be benefits in combined probiotic and antibiotic therapy. Further experiments and future research may be required to support this hypothesis.

DNA sequencing of rpoβ from wild type bifidobacteria that had been exposed to different concentrations of RIF revealed mutations not only on the cassettes but also in regions other than the clusters. This indicates that mutations outside the cassette region also make the bacteria resistant to higher concentrations of RIF. Wild type bifidobacteria also showed resistance to RIF at a concentration of 2 µg/mL. Mutations revealed by DNA sequencing in cassettes 1 and 2 were exactly the same as in MDR *Mtb* rpoβ. Therefore, we can conclude that bifidobacteria is multidrug-resistant since it showed resistance to other antitubercular drugs as well as to RIF. Wild type bifidobacteria showed remarkable and rapid adaptability to RIF when grown in the presence or absence of different concentrations of RIF. The bacteria not only grew normally, but also showed adaptability by varying the rpoβ sequence. Mutations also occurred in regions between the clusters as well as in the rpoβ regions. The mutations outside the rpoβ clusters are novel and have not been previously described.

The adaptability of *B. adolescentis* to RIF was observed when the bacteria were treated with different concentrations of RIF (2, 15, 25 and 100 µg/mL). Large numbers of mutations were observed between cluster regions 2 and 3. There is a unique conserved region, VGEE, between clusters 2 and 3 in *B. adolescentis*. This region was altered to DAAG by exposure to higher concentrations of RIF (100 µg/mL), confirming that mutations in regions other than the clusters lead to multidrug resistance. These variations have not yet been reported in MDR or XDR *Mtb*. Mutations between the cluster regions, therefore, also play a significant role in the adaptable nature of *B. adolescentis*. Mutations in the cluster regions can be considered to generate a certain threshold for RIF resistance, whereas mutations in the regions between the clusters occur above the threshold for RIF resistance. Intrinsic RIF resistance is due to mutations in the cluster regions, and adaptability rises above the threshold RIF concentration. This behavior may be very useful, and it may help to preserve the human microbiome after treatment with RIF, especially in individuals infected with *Mtb*. *B. adolescentis* may thus be the most suitable microbe with Generally Recognized As Safe (GRAS) status for use in combined antibiotic/probiotic therapy. Animal experiments are needed to confirm this hypothesis.

Zhou *et al*.^38^ and Lahtinen *et al*.^39^ concluded that even trace concentrations of common antibiotics, such as vancomycin, chloramphenicol, spectinomycin, β-lactams and RIF, inhibit the growth of bifidobacteria. Genetic characterization of a few streptomycin-resistant bifidobacteria strains have shown that the resistance phenotype is due to chromosomal mutations. Since there is no acquisition of resistance genes, the potential for transferability is negligible. The situation is the same as in the present study. Since the resistance of *B. adolescentis* to RIF is intrinsic and does not involve plasmids, there is no possibility of gene transfer. Kazmierczak *et al*.^40^ showed, however, that the tet (W) gene of *B. longum* was transferable to *B. adolescentis* at low frequencies *in vitro*. This example of gene transfer in Bifidobacterium spp. suggest that it is possible among closely related strains of Bifidobacterium spp. To the best of our knowledge, a similar process of gene transfer to enteric bacteria has not yet been experimentally proved. Although data on antibiotic resistance in bifidobacteria are very scarce, MDR proteins have been identified in *B. breve* and *B. longum*^41,42^. Further research is essential to determine whether antibiotic resistance gene transfer can occur between bifidobacteria and other enteric bacteria.

We used the X-ray structure of RIF for docking calculations. The best docking pose was selected based on the binding energy and visual inspection of the binding pocket. Figure 9a,b and c represent the docked pose, demonstrating the binding interaction of wild-type and mutant proteins with RIF. The data suggest stabilization of RIF at the binding pocket by H-bonding and hydrophobic and ionic interactions. In the case of wild type rpoβ BIFAA, hydrogen bonds were observed with residues Gln-434, Asp-437, Arg-450, Asn-489 and Gln-674 (Fig. 9a). Additionally, hydrophobic interactions between residues Phe-435 and Leu-454 were observed. Ionic interactions in the form of a salt bridge with residue Arg-461 were observed in the wild type protein. In the case of mutant 1, hydrogen-bonding interaction was observed with residues Asp-437, Arg-461, Asn-489, Arg-609 and Gln-676 (Fig. 9b). Hydrophobic interactions were observed with residues Asp-437, Pro-485, Ile-493 and Asn-606 (Fig. 9b). In the case of mutant 2, hydrophobic interactions were observed with residues Gln-434, Phe-435, and Asp-437 and Leu-454 (Fig. 9c). Hydrogen bonding interactions were observed with residues Gln-431, Gln-434, Phe-435, Asn-447 and Arg-450. Significantly, no salt bridge formation was observed in either of the mutant forms of rpoβ BIFAA. Thus, the wild type shows much higher affinity towards RIF due to the favorable energetic interaction of salt bridge formation. Such interaction is absent in both mutant 1 and mutant 2, and they showed considerably less affinity towards RIF. Mutant 1 showed a better affinity for RIF compared with Mutant 2 due to increased hydrophobic interactions. To quantify these interactions and calculate the binding energy, a microsecond molecular dynamics simulation of wild and mutant rpoβ BIFAA would be possible. However, these studies are beyond the scope of present manuscript and will be reported elsewhere. The SDS-PAGE separation of the bifidobacterial lysates (subjected to a higher concentration of RIF) conclude that the bifidobacterial proteins are fluctuating, seven proteins are down-regulated and two up-regulated.

## Materials and Methods

Nine different strains of bifidobacteria (Supplementary Data, Table 1) were obtained from the DSM (Braunschweig, Germany). Cell culture reagents, such as MRS (De Man, Rogosa and Sharpe), Luria-Bertani broth (LB) and *Bifidobacterium* broth and agar, were obtained from Hi-Media Laboratories (Hi-Media Mumbai, India) or Oxoid (Milan, Italy). Antibiotic discs containing ampicillin, kanamycin and chloramphenicol were obtained from Hi-Media Laboratories, Mumbai, India. Specific antibiotics or antitubercular drugs, such as RIF, streptomycin, isoniazid and pyrazinamide, were obtained from Hi-Media Laboratories.

### Growth pattern studies

The growth patterns of the bifidobacterial strains *B. adolescentis, B. animalis* and *B. longum* were investigated at 37 °C. In brief, the bifidobacteria were streaked onto agar plates to obtain individual colonies, and the streaked plates were incubated at 37 °C for 72 h under anaerobic conditions. The resulting colonies were then inoculated into fresh MRS medium (10 mL) and grown at 37 °C without shaking. Later, 4% inocula were subcultured into MRS medium (50 mL) to initiate the growth curve at OD_600_ 0.01–0.05. Bacterial samples were collected every 12 h, and absorbance was measured at OD_600_. The experiments were carried out in triplicate. The growth patterns in the presence and absence of osmolytes were also determined from this stage of culture.

### Analysis of Growth pattern in the presence and absence of osmolytes

From the overnight bifidobacterial cultures, 4% inoculum was subcultured into fresh MRS medium (50 mL), containing either Tween 80 (0.002%) or glycerol (0.05%), for growth curve studies. Samples were withdrawn every 12 h, and absorbance was measured at OD_600_.

### Analysis of Growth pattern in the presence of antitubercular drugs

Bifidobacterial growth patterns in MRS, MRS containing Tween 80 (0.002%) and MRS containing glycerol (0.05%) were recorded in the presence and absence of RIF. The drug was added just before subculturing from stock (20 mg/mL), and care was taken to ensure that the concentration of RIF in the cultures did not exceed 2 mg/mL. The cultures were sampled every 12 h, and OD_600_ values were recorded. The absorbance was then plotted against time.

### Rifamycin uptake

RIF is a red-colored substance with absorbance at 473 nm. Although the procedure introduced by Nokaido *et al*.^43^ is the standard method for measuring the uptake of RIF, we preferred to use HPLC and LC-MS^44^ since this is a very sensitive method and also allows estimation of the approximate molecular weight of substances.

Rough and smooth strains of bifidobacteria were distinguished by agglutination reaction with acriflavin solution (1/1000) and the ability to take up crystal violet^45^.

### Sample preparation for HPLC to determine bifidobacteria adaptability

*B. adolescentis* (OD_600_ = 0.8) was grown in the presence of RIF (480 µg/mL). Samples (50 mL) were withdrawn every 6 h, and the cells were harvested by centrifugation at 5000 rpm for 20 min. The cells were then processed using the procedure described by Bhat *et al*.^44^. Briefly, the washed cells were dissolved in a mixture of methanol: chloroform: water (12:5:3, 3 mL) and heated at 65 °C for 20 min before storing at −20 °C overnight (12–16 h). The samples were then centrifuged, and unlysed cell debris was discarded. The supernatant was collected, vortexed, and centrifuged again at high speed for 20 min. The supernatant was collected and vacuum dried. Finally, the pellet was re-dissolved in ammonium formate (10 mM, 300 µL) at pH 6.3 and extracted with ethyl acetate (600 µL). The organic layer formed by further centrifugation was dried as described above and reconstituted in an HPLC buffer on the day of analysis.

### LC-MS conditions for detection of RIF

High-resolution separation of RIF was achieved using a UFLC liquid chromatography system (Shimadzu, A/B, Rushabh Chambers, Makwana Road, Marol, Andheri (E), Mumbai −400 059) equipped with an LC-20AD pump and an SPD-M20A diode array detector and controlled by LC solution software. Separations were carried out on a Supelco C18 column (5 µm, 25 cm × 4.6 mm) at 25 °C with a guard column prior to the main column to protect against compound accumulation. The pH of the water was maintained at 2.25 throughout the experiment and was adjusted with orthophosphoric acid before mixing with acetonitrile (ACN).

#### Isocratic mobile phase

A mixture of ACN and water (60:40) was sonicated for 15 min using an ultrasonic water bath. The column and guard column were washed thoroughly before the sequential injection of water and ACN. RIF working standards (0.2–100 µg/mL) was prepared by diluting stock solution (1 mg/ml in ACN + 0.02% BHT (Butylated Hydroxy Toluene)) before injection into ACN + 0.02% BHT. The peak areas obtained after injecting a constant volume of solutions containing different concentrations of RIF were plotted against concentration. The concentration of the sample was calculated from the standard curve using the equation Y = mX + C. A graph of the increase in uptake of RIF by the bacteria against time of exposure was plotted.

### MTT assay

All three bifidobacterial strains were grown for 48 h under anaerobic conditions at 37 °C in the presence of different concentrations of RIF. An MTT assay was conducted, as previously described^45^, to distinguish resistance from cell viability. Briefly, 10% MTT (5 mg/mL dissolved in PBS, pH 6.4) was added to drug-containing, drug-free (control) and blank control samples (3 mL). After vigorous vortexing, the samples were incubated for 4 h at 37 °C. The formazan formed was dissolved by the addition of solubilization buffer (20% SDS and 50% DMF *v/v*), and the samples were then incubated at 37 °C for 1 h. The resulting color was measured at 570 nm and compared with the blank control (MRS, MTT and solubilization buffer). The relative OD unit (RODU) was calculated using the following formula, and interpretation of the results was based on the O.D_600_ of the tube being ≥0.1.

$$\mathrm{RODU}=\frac{{\rm{OD}}\,{\rm{of}}\,{\rm{drug}}\,{\rm{containing}}\,{\rm{tube}}}{{\rm{OD}}\,{\rm{of}}\,{\rm{drug}}\,{\rm{free}}\,{\rm{tube}}}$$

RODU values of <0.2, >0.5 and 0.2–0.5 were considered to be susceptible, resistant and borderline resistant, respectively.

The MTT assay was also carried out in 96-well plates to investigate the viability of *B. adolescentis*. Bifidobacterial growth curve studies were then performed, wherein the samples were treated with different concentrations of RIF and samples were collected at 12-h intervals until the stationary phase was reached.

### MS

MS was carried out to detect RIF in chromatography fractions using a Q-Tof Ultima Globa (Waters Ltd., Elstree, UK). The instrument was used in positive ionization mode, with the following operating parameters: spray voltage, 3.7 kV; capillary temperature, 180 °C; analysis range, m/z 300–1000. The m/z peaks obtained were compared with the standard and analyzed using the peak data provided by Pyta *et al*.^27^ and Bhatt *et al*.^44^. A calibration curve was constructed and used throughout the study.

### Determination of Minimum Inhibitory Concentration (MIC)

Serial dilutions of the drugs were made directly in sterile 96-well flat bottom microtiter plates containing MRS medium (100 μL). The RIF concentration range was 0.02–200 μg/mL. The inoculum was freshly prepared, and the OD was adjusted according to the guidelines set by the Clinical and Laboratory Standards Institute (CLSI) to provide CFU of 1 × 10^6^/mL. In addition to wells treated with inoculum (100 mL), the plates included a growth control, a well without drug and a sterile control. The plates were covered with lids and incubated at 37 °C in an anaerobic chamber for 48 h. The absorbance was then measured at 600 nm. The MIC was defined as the lowest drug concentration at which inhibition of bacterial growth was detected, i.e., the OD of the test well is equal to that of the blank well.

### Mutant Prevention Concentration Assay (MPC)

Agar dilution is very commonly used to determine the mutant prevention concentration **(**MPC) since very precise concentration measurements are needed for the validation of MIC data. The MPC assay was carried out using the procedure developed by Sindelar *et al*.^46,47^. Stationary phase cells (1 OD) were harvested by centrifugation at 3000 rpm for 5 min. The cells were then washed and re-suspended in fresh MRS. The cells were serially diluted, and aliquots (10 mL, 10^10^ CFU/mL) that had been previously treated/grown with different concentrations of RIF (0, 0.1, 0.5, 1, 5, 10, 50 and 100 μg/mL) were spread onto MRS agar plates. The plates were incubated at 37 °C under anaerobic conditions for 48 h. Colonies were then counted, and CFU/mL was plotted against RIF concentration. The antibiotic concentration allowed some mutants to be recovered from the susceptible population when the bacteria were spread on plates with different concentrations of antibiotic. The serially diluted samples were also spread onto RIF-containing plates.

### Microscopic analysis of RIF-treated cells

Harvested cells were washed as described previously, and their surface texture/color and morphology were examined using a simple compound microscope and a scanning electron microscope. The same bifidobacteria were again streaked/spotted on RIF-containing plates to determine their antibiotic resistance.

### RIF acclimatization studies

Bifidobacterial cells were grown in the presence of RIF and then harvested, washed, and spotted on agar plates containing different concentrations of RIF. Control bifidobacteria were grown in the absence of RIF, but, after harvesting, they were placed on RIF-containing plates. This procedure demonstrates the ability of bifidobacteria to adapt to different concentrations of RIF.

### PCR amplification of different bifidobacterial RIF pockets (rpoβ)

Bifidobacteria showing resistance to RIF were subjected to PCR amplification of specific regions of the rpoβ gene (RIF pocket). Genomic DNA (gDNA) was isolated from all eight strains of bifidobacteria. The relevant bacteria were grown at 37 °C under anaerobic conditions for 36 h using a Gene JET genomic DNA purification kit (Thermo Fisher Scientific India PVT.LTD.403–404, Delhpi B’ Wing, Hiranandani Business Park, Powai, Mumbai-400076), according to the manufacturer’s instructions. gDNA (100 ng) was used as the template for the PCR (100 μL). The reaction mixture contained MgCl_2_ (1.5 mM), dNTPs (0.2 mM), primers (20 pmol) and DyNazyme II polymerase (2 units) in 1 × buffer. The rpoβ forward primer and reverse primers used for the amplification were 5′GTGTGGTCCGCGAACGTATGA3′ and 5′AGGATGACGTCGCCGGAAT3′, respectively. The PCR amplification parameters were as follows: initial denaturation at 94 °C for 4 min, followed by 35 cycles of denaturation at 94 °C for 30 s, annealing at 56 °C for 45 s (for strains 10140, 20219 and 20083) and extension at 72 °C for 1 min. The final extension was carried out at 72 °C for 4 min. Finally, all amplifications were subjected to 2% agarose gel electrophoresis.

### DNA sequencing of wild type and rpoβ (RIF pocket) mutants (mutant 1 and mutant 2)

After completion of the growth curve studies in the presence of different concentrations of RIF, the cells were harvested and gDNA was extracted. The gDNA was then used for amplification of the RIF pocket gene, as described above. Agarose gel electrophoresis was used to separate the amplified products. The amplicons were further purified and subjected to DNA sequencing.

### Isolation of bifidobacterial membrane proteins

The procedure described by Mattarelli *et al*.^48^ was used with slight modifications. Bifidobacteria were grown at 37 °C under anaerobic conditions. One set was grown as the control, with no added antibiotic, and the other was the test set. The cells were grown until the OD_600nm_ reached 1.8 after subculturing from a culture grown for 36 h. The cells were harvested by centrifugation at 14000 rpm at 4 °C for 15 min and washed three times with buffer (PBS + PMSF). The cells were then dissolved in PBS + PMSF buffer (2 mL) and sonicated at 30% amplitude, with a 30 s on/off cycle, until complete cell lysis was observed. Total cell protein was processed as described by Mattarelli *et al*.^48^. The total protein content in the supernatant was measured, boiled with sample buffer and separated on 12.5% Tricine SDS-PAGE.

### Scanning electron microscopy studies

Scanning electron microscopy (SEM) was used to investigate the changes in surface morphology of *B. adolescentis* and *B. longum* grown in the presence and absence (control) of RIF. The cells were harvested as described in the section on growth curve studies. Bifidobacteria that had been grown and harvested were washed twice with phosphate buffer. The pelleted cells were treated with 2% glutaraldehyde and incubated at 4 °C overnight (12–14 h). The cells were then washed using a 10–100% ethanol gradient. The cells were finally re-suspended in absolute alcohol (50–100 µL). An aliquot (~2 µL) of the sample was placed on a cover slip, desiccated and observed using SEM. Only appropriate and convincing images, with 20,000x magnification, were considered.

### Homology modeling

The amino acid sequence (1186 residues) of the target protein, DNA-directed RNA polymerase subunit beta (UniProt code: *A1A317 (*rpoβ *BIFAA)*), was retrieved from the UniProt protein sequence database (http://www.uniprot.org). The three-dimensional model was constructed using a comparative modeling strategy with the template structures of RPOB_MYCS2 (PDB code: 5VI8) and RPOZ_MYCTU (PDB code: 5UH5). Sequence alignment was derived from multiple mapping methods as implemented in the M4T comparative protein modeling tool^49^. The template file generated was used as input to generate homology models by the derivation of restraints from the given related structures and their alignment with the target sequence using comparative homology modeling as implemented in Modeller 9v1^50^. The same methods were used for generating homology models of the two mutant proteins, mutant 1 and mutant 2. All computational models were run with the macOS Sierra operating system (v 10.12.3) with Intel Core i5 3.3 GHz processor with 8 GB of RAM.

### Docking of rpoβ BIFAA and mutant proteins with Rifampicin

The binding pose of RIF into rpoβ *BIFAA* was achieved using AutoDock 4.2, which is widely used software for molecular docking studies^51^. We used the default rigid protein residues in the docking simulation. The x-ray structure of RIF in complex with the *Mycobacterium tuberculosis* transcription initiation complex was used in docking to rpoβ BIFAA^52^. The ligand RIF was kept flexible to explore an arbitrary number of torsional degrees of freedom spanned by the translational and rotational parameters. AutoDock4.2.6 calculates the binding energies between protein and ligand using atom affinity potentials, which are pre-calculated on grid maps using Autogrid4. A grid box of dimensions (60 × 60 × 66) in the X, Y, and Z directions, with a spacing of 0.375 Å, was used dock RIF into the active site of rpoβ *BIFAA* in all of the conformations. Docking was performed using the Lamarckian Genetic Algorithm (LGA) in the conformation space defined by the grid. The LGA parameters were of population size: 150, mutation rate: 0.02 and crossover: 0.8. The maximum number of energy evaluations was set at 2.5 million, and the maximum number of generations was kept 27000. The RIF confirmations were ranked in the order of increasing docking energies. The top poses were selected from each docking simulation. Further, the best pose was selected based on binding energy. This confirmation was regarded as the binding conformation between rpoβ *BIFAA* and RIF. Interactions were analyzed using AutoDock Tools and PyMol^53^.

### Disorder analysis of rpoβ BIFAA

To predict disordered regions in rpoβ BIFAA, secondary structure analysis was performed using the program PSIPRED^54^. The analysis revealed that the protein consists of helix and strands. Beginning and ending residues mainly shows disorder characteristic (Ramachandran plot, data not shown).

## Electronic supplementary material

Supplementary Table 1
