# Supplementary material for: Bifidobacterium adolescentis is intrinsically resistant to antitubercular drugs
Source: Sci Rep. 2018 Aug 9;8:11897. doi: 10.1038/s41598-018-30429-2 (PMC6085307; doi:10.1038/s41598-018-30429-2)
Supplement: Supplementary file 1 — Supplementary Table 1 [file 41598_2018_30429_MOESM1_ESM.pdf]

Title: *Bifidobacterium adolescentis* is intrinsically resistant to antitubercular drugs

Authors: Dhanashree Lokesh[1], Raman Parkesh[2] and Rajagopal kammara[1]

**Bacterial strains**	**Strain No.**	**Reference**
B. animalis Subspp *lactis*	DSMZ 10140	Meile *et al*. 1997
B. thermacidophilum Subspp *thermacidophilum*	DSMZ 15837	Dong *et al*. 2000
B. adolescentis	DSMZ 20083	Reuter 1963
B. longum Subspp *infantis*	DSMZ 20088	Reuter 1963
B. longum Subspp *longum*	DSMZ 20219	Reuter 1963
B. asteroids	DSMZ 20089	Scardovi and Trovatelli 1969
B. animalis	DSMZ 20105	Mitsuoka 1969
B. breve	DSMZ 20213	Reuter 1963
B. indicum	DSMZ 20214	Scardovi and Trovatelli 1969
B. catenulatum	Fecal isolate 14 month old baby	This study

Supplementary Table 1: Strains used in this study
